# Non-medical facilitators and barriers towards accessing haemodialysis services: an exploration of ethical challenges

**DOI:** 10.1186/s12882-018-1140-x

**Published:** 2018-12-03

**Authors:** Godwin Pancras, Judith Shayo, Amani Anaeli

**Affiliations:** 1 Department of Bioethics and Health Professionalism, Muhimbili University of Health and Allied Sciences, P.O Box 65001, United Nations Rd, Dar es Salaam, Tanzania; 2 Department of Clinical Nursing, Muhimbili University of Health and Allied Sciences, Dar es Salaam, Tanzania; 3 Department of Development Studies, Muhimbili University of Health and Allied Sciences, Dar es Salaam, Tanzania

**Keywords:** Chronic haemodialysis, Access, End-stage renal disease, Ethical challenges, Barriers, Tanzania, Developing countries, Africa

## Abstract

**Background:**

Like most of the sub-Saharan countries, Tanzania faces significant increase in the number of patients diagnosed with an end-stage renal disease (ESRD) among which only a few manage to receive chronic haemodialysis services (CHD). Yet little is known about the non-medical facilitators and barriers towards accessing these services and the associated ethical challenges.

**Methods:**

A phenomenological study design which employed a qualitative approach was used. The study was conducted at the dialysis unit harboured within Muhimbili National Hospital. Data were collected from purposively sampled health care providers and ESRD patients by using in-depth interviews. Text data obtained were analysed based on inductive and deductive content analysis methods to formulate major themes.

**Results:**

Fourteen key informants were interviewed including nephrologists, renal nurses, social workers, nutritionists and ESRD patients. Three major themes were formulated: a) non-medical facilitators towards accessing CHD services which enshrines two sub-themes (membership to health insurance scheme and family support), (b) non-medical barriers towards accessing CHD services which enshrines four sub-themes (affordability of treatment costs, geographical accessibility, availability of CHD resources and acceptability of treatment procedures) and lastly (c) ethical challenges associated with accessing CHD services which also enshrines three sub-themes (dual role of health care providers, patients autonomy in decision making, and treatment disparity).

**Conclusion:**

Non-medical facilitators to access CHD benefits few patients whereas non-medical barriers leave many ESRD patients untreated or partially treated. On the other hand, ethical challenges like treatment inequality are quickly gaining momentum. There is a need for guideline highlighting importance, position, and limitation of non-medical factors in the delivery of CHD services in Tanzania and other developing countries.

**Electronic supplementary material:**

The online version of this article (10.1186/s12882-018-1140-x) contains supplementary material, which is available to authorized users.

## Background

Before the invention of dialysis treatment in 1960s, suffering from an end-stage renal disease (ESRD) was considered a slow inescapable death sentence [1, 2]. Even after initiation of haemodialysis treatment, it was not made available to every patient due to the scarcity of personnel and machines. But it was made available only to those with acute kidney failure to avoid death and to non-diabetic, fit and young patients who could easily be treated and return to their normal condition with less demand of the already limited resources [3]. By 1960, Belding Scribner and his colleagues discovered a shunt which helped long-term dialysis of ESRD patients [4]. The discovery held back the notion that dialysing ESRD patient is “unethical” or “cruel” [5]. The lifespan and quality of life of ESRD patients can now be extended but is it the same for developing countries like Tanzania.

Among the three types or modalities of renal replacement therapy: haemodialysis, peritoneal dialysis and kidney transplantation; haemodialysis (HD) is the most preferable option for many developing countries [6]. Patients receiving chronic haemodialysis (CHD) treatment are dialyzed three times per week with each dialysis session lasting up to four hours [7] during which excess body fluid and toxic components such as urea are removed out of the body [6]. On the other hand, peritoneal dialysis especially Continuous Ambulatory Peritoneal Dialysis (CAPD) has received varied acceptance among African countries due to high costs of peritoneal fluids [8, 9]. Furthermore, high rates of infection among patients using peritoneal dialysis raises a concern for those in Africa where most of the people still reside in unhygienic environments which lack clean water, low electric supply and limited sanitary education [10]. Although most of the developing countries including Tanzania view CHD as a cheaper alternative compared to other types of renal replacement therapy but majority of ESRD patients remain untreated [10].

According to Moosa and Kidd, access to CHD services is largely determined by social factors than medical factors [11]. For this study, the term access is not merely an entry into the health system but also an opportunity to use dialysis services and have patients’ needs for services satisfied [12]. Most of non-medical factors that hold ground in Africa when determining adequate ESRD patients for CHD are implicit or inconsistent and have been abandoned by most of the developed countries [13]. In Tanzania, a negligible number of patients receive CHD services, up to 2015 at least 267 patients were on CHD services [14]. Although the actual data on the prevalence of ESRD among Tanzanians is scarce, more than 83.7% of adult diabetic patients have renal disease which marks them as possible victims for ESRD [15]. Given the evolving demographics of ESRD population, the annual increase of ESRD patients and increasing costs of CHD treatment in Africa, non-medical facilitators and barriers experienced by ESRD patients have to be explored and the ethical challenges have to be made explicit. This necessitated this study to be conducted.

## Methods

The study employed the phenomenological study design with qualitative approach [16] to explore non-medical facilitators, barriers and associated ethical challenges during access of chronic haemodialysis (CHD) services at Muhimbili National Hospital. Muhimbili is the largest and only government-owned hospital offering haemodialysis services in Dar es Salaam. It has a total of 16 working dialysis machines. Generally, 75% of all haemodialysis services in Tanzania are offered in Dar es Salaam region [17].

During the two month period (April to June 2017) data were collected and analyzed simultaneously. Data were extracted from two purposively sampled groups of key informants [18]: Healthcare providers and patients. The major selection criterion for health care providers was at least 2 years of work experience at the dialysis unit whereas for ESRD patients at least 3 months of dialysis. There are reasons for not interviewing ESRD patients not receiving CHD treatment. The main one being the absence of contact details at the unit and none existence of national ESRD repository for none dialysed patients. Additionally, the chance of finding such patients alive in a hospital ward was slim due to death or discharge. Unlike their counterparts, patients receiving CHD are suitable to elicit experiences and hurdles they go through to receive dialysis, they also offer an inside experience on how treatment is provided at the unit. Their views were further harmonized by the health social worker who deals with social concerns for patient’s access to dialysis. The social worker was interviewed twice for clarity on how patients are enrolled in CHD treatment.

During patients’ selection, a list of names was obtained from the nurse in charge with the permission from the head of the dialysis unit. The list highlighted the time for dialysis and day of patient attendance. On each day of the interview, patients were approached by the researcher and voluntarily asked to participate in the study. Those who agreed to participate were free to choose to be interviewed before or after dialysis sessions. Most of the patients chose to be interviewed prior to commencing the dialysis due to the fact that after dialysis they feel weak and lightheaded. For the healthcare providers, names and contact details were obtained from the head of the dialysis unit. Some of them were contacted physically and others were called to ask for their participation. Those who agreed were free to decide on the time, date and place of interview.

Each group was assigned to its own interview guide (Additional file 1). The interview guides where pilot tested with few patients and a healthcare provider and questions rephrased. Each interview guide had three open-ended questions with subsequent probing questions. The questions were tailored to answer the three study objectives regarding non-medical facilitators, non-medical barriers and ethical challenges towards accessing CHD services at Muhimbili National Hospital. Interviews were conducted by the researcher in a separate and an isolated room away from the earshot of the healthcare providers and other patients. This was done so as to maintain the privacy of key informants and confidentiality of the information shared with the researcher. Additionally, the researcher ensured that questions are fully exhausted by participants and data collection stopped when all the themes became saturated. With permission from key informants, interviews were audio recorded. Furthermore, field notes were taken for each interview to assist the analysis.

### Qualitative data analysis

Content analysis was used to analyse data guided by deductive and inductive approaches. The rationale for using the two approaches based on the desire to minimise weaknesses which are visible once a single approach is used [19].

With the aid of F4 version 3.0.3 computer program, the audio taped data were transcribed verbatim into a Microsoft word document by the researcher. All transcripts were translated from Swahili to English language by the bilingual expert. Then the transcripts were read and re-read to familiarize and gain more understanding of the concepts portrayed by key informants. The identified concepts were then coded by using pre-determined codes and emergent codes. Pre-determine codes were obtained from various studies centred around barriers and access to health care services or CHD services in developed and developing countries [17, 20–27]. For the information which did not fit within the pre-determined codes, a new code was formulated to accommodate them. Both emerging and pre-determined codes were grouped into 9 meaningful clusters named as sub-themes. Due to the differences and similarities, sub-themes were further reduced into 3 main themes namely: non-medical barriers towards accessing CHD services, non-medical facilitators towards accessing CHD services and ethical challenges associated with accessing CHD services. The three sub-themes: availability of CHD resources, acceptability of treatment procedures and treatment disparity were not formulated from existing literature but from text data during analysis. Although it clearly extrapolated the strength of using both inductive and deductive content analysis methods, the process of data collection and analysis was iterative.

Moreover, the whole process of assigning texts into codes, assigning codes into sub-themes and grouping sub-themes into main themes was systematically and logically made with inference to the coding scheme which was modified whenever new information arose. Due to a large volume of text data NVivo version 10 computer software was used to organize text data into respective codes and themes.

## Results

### Description of study participants

A total of 14 respondents (8 male and 6 female) were interviewed, including 2 nephrologists, 1 nutritionist, 1 social worker, 4 renal nurses and 6 patients with end-stage renal disease (ESRD) receiving chronic haemodialysis (CHD) services at the hospital’s dialysis unit. The sample size was limited by saturation [28] as redundant information from subsequent respondents began to emerge. Among all patients interviewed, 3 did not have health insurance, 2 were unemployed, 3 had a university education and 3 have been receiving CHD services for at least one year. The age of all key informants ranged between 21 and 60 years old.

### Presentation of findings

Three major themes emerged: (a)non-medical facilitators towards accessing CHD services, which enshrines two sub-themes [membership to a health insurance scheme and family support]; (b) non-medical barriers towards accessing CHD services, which enshrines four sub-themes [affordability of treatment costs, geographical accessibility, availability of CHD resources and acceptability of treatment procedures]; (c) ethical challenges associated with accessing CHD services, which also enshrines three sub-themes [dual role of health care providers, patient autonomy in decision making, and treatment disparity].

### Non-medical facilitators towards accessing CHD services

#### Membership of health insurance scheme

Key informants reported that having health insurance did not only reduce the cost burden but also ensured timely access to CHD services for insured patients. Uninsured patients had to first undergo socio-economic assessment by social workers before commencing treatment as a result, creating a time lag.

***“****…you find that it is easier for the insured patients to get services than the uninsured patients. It is easy because expenses are paid by the insurance but the uninsured patients have first to go to social workers …****”*** (Healthcare provider 08).

Further, the appointment schedule for dialysis was solely determined by whether or not a patient is insured. With the dialysis unit conducting four dialysis sessions per day: insured patients were scheduled to attend morning and afternoon dialysis sessions whereas uninsured patients were scheduled to attend evening and night dialysis sessions. The challenge for the patients scheduled to attend evening dialysis sessions was the complications of going back home; as a result, they had to sleep on patient-waiting benches outside the unit until morning or opt to return home without being dialyzed.

*“…those who enter into dialysis in the evening some sleep here [dialysis centre]. If he/she finishes [dialysis] let us say at 1 or 2 am and has no private transport. He/she has to sleep here…”* (Patient 03).

That being the consequence, to some it is unbearable. As illustrated by one patient:

*“If the patient enters into dialysis at 10 pm…at what time will he/she finish dialysis? So some decide to go back home without being dialyzed.”* (Patient 01).

However, one key informant explained that the difference in appointment schedule among dialysis patients was due to influence from health insurance companies.

*“…came an issue of insurance companies complaining about their clients [patients] being mistreated… so it came out that insured patients should be given priority, and should be scheduled for morning and afternoon dialysis sessions.”* (Healthcare provider 03).

#### Family support

Key informants in particular healthcare providers reported of family readiness to support the patient as one among the major considerations for receiving CHD. Cases of patients being abandoned by their family members even their spouses were also extrapolated by key informants. This abandonment can be due to financial difficulties or family problems. One patient explained that:

*“…there are also family financial problems which can prevent a person from getting these [dialysis] treatments…you also have to pay for lab tests”* (Patient 06).

Unfortunately, sometimes money may not be a problem instead family members are challenged in making decisions whether their patient should pursue dialysis or not and this created a time lag. One healthcare provider portrayed that;

*“Sometimes families take a long time to make decisions to accept dialysis treatment…”* (Healthcare provider 04).

Moreover, the importance of family support was agreed among most of the participants. Thus family support is not necessarily financially but also emotionally and psychologically. Unsupported patients would be stressed and fill discriminated.

*“I expect family members to motivate me, be friendly to me so that I do not feel discriminated.”* (Patient 05).

Additionally, in determining who will be the potential donor, family support was a requisite.

*“…when you involve the family it determines who will donate a kidney … [t]hat is the most important thing.”* (Healthcare provider 07).

### Non-medical barriers towards accessing chronic haemodialysis (CHD) services

#### Affordability of treatment costs

During the interview key informants reported how expensive CHD services are. If a patient is unable to pay for dialysis services even after cost subsidization he/she will be disqualified from receiving CHD.

*“If you have not paid how will you enter into dialysis? You have to go pay and bring the receipt to be registered for dialysis.”* (Patient 06).

Pooling out whatever family resources available were common among patients striving to raise money for CHD. The above key informant lamented that;

*“If you have land or livestock you are forced to sell for your patient to receive treatment. People sell even their houses; they don’t sell them for pleasure but because of problems…”* (Patient 06).

Having patients who cannot pay for dialysis expenses or patients who depend on cost subsidizations and exemption entailed a huge financial load to the Hospital and the solution was to keep that number low. And this was a common thought for health care providers and patients as quoted below.

*“…it is a big financial load to the unit [dialysis unit]… this dialysis unit [Muhimbili dialysis unit] is running on losses because of the exemption system.”*(Healthcare provider 04).

*“If they allow us to do dialysis without paying, how will they buy other machines or repair failing machines. That is why they have to consider patients who can pay first…”* (Patient 05).

Moreover, to extensively reduce treatment costs incurred by the dialysis unit, patients under exemption received simply dialysis with no other consumables like drugs.

*“…for them [public patients] they are just given a favour to pay that thirty thousand Tanzania shilling [˷14 US Dollars] and do dialysis but buying other medication is upon them.”* (Healthcare provider 03).

#### Geographical accessibility

A large distance between the patient’s household and the dialysis unit influenced patients to skip haemodialysis sessions, rearrange appointment schedules or completely abandon CHD. Patients residing outside Dar es Salaam region were most likely to be denied CHD.

*“…for you to enter into dialysis you must assure us of your accommodation here in Dar es Salaam… and they have to be trustworthy…”* (Healthcare provider 06).

The reason for the above judgement could have been influenced by what was clearly extrapolated by patients themselves. One patient said that:

*“…someone can be assigned to come three times a week but he just cancels and comes twice a week due to … the distance he has to travel to come for dialysis.”* (Patient 01).

The only way of acquiring accommodation in Dar es Salaam was for patients who do not have relatives to rent houses. But sadly finding affordable places near the dialysis unit seemed difficult. So, patients have to look for cheap places which are far from the dialysis unit which require them to pay for transportation fee to reach the dialysis unit.

*“They cannot rent places near Muhimbili National Hospital because it is too expensive so they rent at Magomeni or Kimara [sub-urban area] and other places where renting are cheap… another issue is on transport…”* (Healthcare provider 05).

#### Availability of CHD resources

On average key informants reported of fifteen to seventeen machines working day and night to cleanse out toxins from the body of end-stage renal disease (ESRD) patients. This resulted in a breakdown of some of the machines, and when that happened some patients had to wait for other patients on operating machines to finish their dialysis sessions or opt to return home.

*“…if on that day the machine is not working or the machines are few…obvious you have to wait or go back home”* (Patient 03).

Apart from the limited availability of haemodialysis machines, limited availability of health care providers, such as renal experts including nephrologists was also extrapolated. Stressing on the negative consequences of having many patients with a limited number of renal experts, one key informant said that:

*“…find many patients in need of my services but I cannot satisfy all of them…so I fail to take care of them as it is required…”* (Healthcare provider 07).

Moreover, key informants linked the case of patient overload with how CHD services are redistributed in the country:

*“… the shortage of dialysis centres and services being centralized at one place makes you as a doctor to be overloaded receiving a lot of patients whereas the services could also be provided at Amana, Temeke [nearby hospitals] or Lindi and Mtwara [up-country regions]”* (Healthcare provider 05).

#### Acceptability of treatment procedures

The way in which chronic haemodialysis treatment is delivered was reported to profoundly affect the preferences or attitudes of some patients. Based on religion and culture some patients refused to be dialyzed despite being well informed about the nature of the disease and treatment modalities available. Taking blood out of the body, cleaning and re-taking it back to the body contradicted the religious beliefs of some patients. On the other hand key informants reported of family members or patients themselves appealing to religious prayers as a cure for the end-stage renal disease (ESRD).

*“Most of the people based on their beliefs either cultural or religious refuse dialysis saying that we will just go to the church and the kidneys will heal themselves…”* (Healthcare provider 04).

Patients relying upon such beliefs contradicted the health care providers’ duty to avert death.

*“A patient said according to his religious beliefs…blood is not something you should remove outside the body….since he was the one receiving treatment, without his consent nothing can be done… later the patient died.”* (Healthcare provider 06).

### Ethical challenges associated with accessing chronic haemodialysis (CHD) services

#### Dual role of health care providers

Key informants reported of health care providers acting to fulfil the wishes and interests of their patients whereas on the other side being required to ensure proper utilization of institutional resources which were meagre. Such conflicting interests left health care providers feeling guilt especially when they had failed to save the patient’s life provided that it is the core obligation of their profession.

*“I have the patient and am supposed to help him/her to get the CHD services, but also I am the hospital employee…but you will be limited because some types of equipments are supposed to be bought outside the hospital or available at the hospital but it isnot for free…so you can enter into conflict with the hospital…because at the end of the day nobody is going to return the money for the equipment, the hospital has to find a way to cover it.”* (Healthcare provider 06).

The key informants also lamented that, the difficulty in deciding whether a patient should or should not receive chronic haemodialysis was contributed by lack of financial support from the government. The government does not reimburse treatment expenses for patients being treated under the exemption scheme; the whole burden is left to the hospital dialysis unit.

*“…when you provide services to a large number of public patients [patients under exemption] the government does not provide any money to pay back to the hospital so the hospital itself has to find out how to raise income to cover the treatment expenses. So if a large group of patients is uninsured it means they [Muhimbili National Hospital] will use a lot of money to treat them and later will fail to run itself.”* (Healthcare provider 02).

#### Patient autonomy in decision making

When making decisions healthcare providers had to educate not only the patients but also their caretakers on the nature of treatments, benefits, risks and what is required during and after the commencement of CHD. Patients had a final say regarding what, how or when their illness should be attended.

*“…if the patient is conscious he is always involved, he is told about his health problem, what he has to do, they explain to him all the procedures. At the end of the day, he has to sign the form…”* (Patient 03).

Some of the ESRD patients, who came late to the dialysis unit, seriously ill and unconscious, were directly dialyzed under the auspices of their caretakers. But that did not exclude the patient from deciding whether or not to continue with the treatment after their health condition has stabilized, as portrayed below:

*“…if your condition is worse you are not told [involved in making decisions], your caretakers are the one told. Caretakers can even be given a form to sign on your behalf.”* (Patient 01).

*“When you regain your consciousness it is when you are educated about dialysis treatment”* (patient 03).

However, the decision of either to start or continue with CHD treatment was overridden when the patient was deemed unable to pay the required expenses even after the cost subsidization. Depending on economic assessments done by social workers, treatment costs were waived up to less than 50 % of the normal treatment cost whereby a patient receives dialysis only. Thus some patients who wanted to be treated had to remain home until they raise money to pay for CHD.

*“…others [patients] can continue to receive the services [haemodialysis] only for the days which they can pay…”* (Healthcare provider 02).

Moreover, when healthcare providers made decisions as to whether a patient should or should not receive CHD, family members were also asked to participate. For that reason, some family members ended up deciding to take patients back home or to other places aligned with their beliefs.

*“A patient…said that everything to do with my treatment have to be decided by my wife… his wife said I will take him for prayers”* (Healthcare provider 06).

#### Treatment disparity

Key informants reported of patients being offered different treatment options according to their financial status. The number of dialysis sessions, appointment time, kinds of medication prescribed depended on whether a patient is insured, paying out of pocket or under exemption (public patients). Patients who were uninsured yet paying under exemption received not more than two dialysis sessions per week whereas insured patients received three dialysis sessions per week.

*“…we do dialysis two times a week because we don’t have health insurance unlike those with health insurance who do three times a week”* (Patient 05).

The difference was also evident in the prescription of drugs like EPO (erythropoietin) and arrangement of dialysis schedules. Most of the patients under exemption resort to less expensive drugs or taking no drugs at all in order to reduce treatment expenses. But also, such patients were scheduled to attend evening and night dialysis sessions.

*“Public [uninsured under exemption] patients do not receive medications, they do not get EPO [a drug for red blood cell production], and most of their [public patients] appointments are scheduled in the evening or at night.”* (Healthcare provider 03).

The above participant added that:

*“… If the insured patients fall sick they are prescribed and given medication, but that is not done if the uninsured patients fall sick.”* (Healthcare provider 03).

## Discussion

Receiving chronic haemodialysis (CHD) treatment as a means to combat the carnage of end-stage renal disease continues to be a challenge in Tanzania and Africa as a whole. This qualitative study focused on exploring non-medical facilitators and barriers towards accessing CHD and associated ethical challenges at one of the referral government-owned hospital in Tanzania. Three major themes were explored: non-medical facilitators towards accessing CHD services, non-medical barriers towards accessing CHD services and ethical challenges associated with accessing CHD services.

Possession of health insurance emerged as a major facilitator through which a patient can timely and conveniently access CHD at the dialysis unit. Thus for insured patients, it was easy to receive CHD than uninsured patients. Unfortunately, in Tanzania, access to health insurance remains confined to the people employed in formal sectors and wealthy individuals. Also, most the health insurance companies largely operate in urban areas [29]. Rural population employed in the informal sector are covered by the Community Health Fund (CHF) which cannot cover any referral costs beyond district level. Sadly, almost all dialysis services are provided in referral hospitals which appear to be above what CHF can cover. This leaves patients coming from rural areas and patients with deprived economical status vulnerable to the sufferings of end-stage renal disease (ESRD). Also, inconvenient dialysis schedule leaves uninsured patients with two options at hand: to quit or postpone treatment. Quitting or postponing haemodialysis for ESRD patients entails inescapable death. The undue influence from insurance companies which forces the rearrangement of dialysis schedules at the dialysis unit in order to suit their clients (insured patients) is a result of competition in the healthcare market. The findings are supported by Miller who reported that, the ability of patients to obtain appointments at the time they desire is highly controlled by an aspect of competition in health care [30]. This implies failure of the government to implement universal access as a result implicating the survival of unhealthy and uninsured patients at the dialysis unit.

Additionally, family readiness to support their patient was a facilitator to easily and timely access CHD. For CHD being a long-term and lifestyle changing treatment, patients become economically dependent on their families and to family resources. However, there are times when families abandon their patients and this causes psychological disturbance to patients. Similar findings have been reported by Thong et al.*,* who noted that dialysis patients who perceived of not receiving enough social support be it from family members, spouse and colleagues had high mortality out of which lack of daily emotional support contributed up to 10% increase in mortality [31]. If family members are not empowered enough to care for their patients receiving CHD, efforts of health care providers to restore the patient’s medical condition using scarce resources available is likely to be wasted.

Non-medical barriers towards accessing chronic haemodialysis (CHD) were also explored. Key informants named overwhelming costs as a major barrier in receiving CHD services whereby a patient has to pay almost three hundred thousand Tanzanian shillings (136 US Dollars) for single haemodialysis session, that is if the patient is not in the exemption payment system. Given that more than one-third of Tanzanians earn less than 1$ per day it is clear that, without exemption, most of the patients will never afford the current CHD services [17]. Similarly, this is evident in many developing countries especially in sub-Saharan Africa where more than 90% of ESRD patients remain untreated [10, 32]. CHD being among the most expensive yet long-term treatment, paying out of pocket is likely to make the financial status of ESRD patients worse than a normal population. For that reason creating a new circle of poverty exacerbated by increased health care expenses.

The patient’s geographical location especially distance from the dialysis centre also emerged as a barrier for ESRD patients to access CHD. It is a barrier with a geographical bias for ESRD patients of the same country. Patients without relatives in Dar es Salaam have to rent places to stay near the hospital as a result, incurring rent costs. Renting a house raises a concern of whether patients are committed to undergo haemodialysis for the rest of their lives. Renting a house implies being far from family members and friends. Renting a house creates additional increase in treatment expenses compared to the patients dwelling in Dar es Salaam. With such emotional and financial difficulties many patients will fail to carry CHD treatment in a long term. Some patients despite renting or staying with their relatives still travelled more than 37 miles to reach the dialysis unit; this threatened the increase in mortality risk. Similarly, Tonelli et al.*,* found out that, haemodialysis patients who stayed more than 31 miles away from the dialysis unit had higher mortality compared to patients who stayed less than 31 miles to the dialysis unit [33]. Similar findings have also been reported by Thompson et al., and Moist et al., [26, 34]. Patients residing outside of Dar es Salaam or in regions without dialysis centres are forced to travel to Dar es Salaam or accept death as their fate. Such unnecessary deaths could be avoided if dialysis centres were increased and distributed evenly throughout the country.

Additionally, limited availability of chronic haemodialysis (CHD) resources emerged as a barrier hindering access to CHD services. The number of renal experts including nurses and nephrologists was surprisingly too small and overwhelmed by the daily increasing number of end-stage renal disease (ESRD) patients in the unit. Similar findings have been reported by Katz et al., who noted that the number of nephrologists compared to the general population was less than 1 per million population (pmp) or even absent in most African countries [21]. Such overload hinders adequate prescription of treatment since doctors not often see their patient or spend time with them. Moreover, if the shortage of haemodialysis machines persists, the opportunity to obtain haemodialysis treatment remains slim, appointment schedules frequently rearranged and length of stay at the unit waiting for dialysis increased.

Regarding the acceptability of dialysis treatment procedures, some patients believed that prayers are enough to heal the kidney and relieve them from the sufferings of ESRD. Similar findings have been reported in a few studies about ESRD patients considering religion to be a healing factor whereby having faith in God is a cure for their illness [35, 36]. Given that, it is from cultural and religious beliefs that people find the meaning of life and comfort, care models highlighting the integration of religion or cultural beliefs and care for end-stage renal disease ought to be established.

Apart from non-medical facilitators and barriers, ethical challenges associated with accessing CHD services have also been explored: the dual role of health care providers, respect for patient autonomy in decision making and treatment disparity. Health care providers found themselves uncomfortable when they had to play a dual role that is as a gatekeeper to hospital resources and at the same time to advocate patient’s interests. ESRD being a potential killer disease most patients suffering from it would opt to be treated and more surprisingly to be treated for free. If that happens, with the current limited financial support from the government, the dialysis unit is most likely to collapse. For that reason, health care providers act with caution in making sure that the hospital incurs minimum financial losses and if possible none. For healthcare providers not being able to fulfil their oath core obligations [37], leaves them feeling guilty and blameworthy. This is supported by a study conducted among Norwegian general practitioners which found out that it was difficult to refuse treating a patient in front of them instead they referred patients to third parties or they appealed to available guidelines to make decisions [38]. But for the country like Tanzania whose government is still reluctant to make treatment against ESRD a priority, most of the healthcare providers are destined to remain conflicted yet uncomfortable with the gatekeeper role of institutional or social resources.

Also respecting patient autonomy in decision making was a challenge not only to health care providers but also to patients. Decisions made by a patient can be overruled if the patient fails to financially support their own treatment. This subjects the patient’s autonomy into jeopardy. Moreover, it calls for more evaluation and understanding of boundaries for the patient’s autonomy. But financial ability as a boundary to patient’s autonomy seems inescapable with the current commoditization of health care services, CHD being one. Rowe and Moodley express their concern about the amendments to the South Africa National Health Act which ought to recognize patients legally as consumers that is.

“*If the patient is considered a ‘consumer’ of healthcare…the doctor takes on the role of ‘provider’ or ‘supplier’ of the ‘commodity’ or ‘product’ of health care. This role-shifting could result in the replacement of professional ethics with marketplace or business ethics*” [39].

If the autonomy of ESRD patients seeking CHD remains financially handicapped, only the needs of wealthier patients will be taken care of.

Also, key informants reported about treatment disparities and privileges between health insured patients and uninsured patients paying under the exemption. The disparities were evident in the number of dialysis sessions, medication prescribed and appointment schedules. Uninsured patients are only dialyzed twice a week compared to insured patients who are dialyzed thrice a week. This difference in dialysis sessions mirrors the differences in removal of toxins and other body wastes. This means that uninsured patients who received few dialysis sessions per week could be left with a higher level of toxins compared to their counterparts. Similarly, a cross-sectional study involving patients on haemodialysis conducted in Iran found out that the required urea clearance level is likely to be achieved with more dialysis sessions [40]. Likewise, Chowdhury et al.*,* noted that limiting the number of dialysis sessions leave most patients displaying uremic symptoms, this results in heightened mortality and morbidity risk [41]. Apart from the difference in dialysis sessions, medications like Erythropoietin (EPO), the highly recommended drug for haemodialysis patients with the anaemic condition are made available to uninsured patients only if they can pay for it. If CHD services are not fully funded through health insurance, the everyday widening gap of inequality between ESRD patients is there to stay.

No research if any goes without limitations; thus due to qualitative nature, the study cannot be generalized to other dialysis units inside or outside of Tanzania due to context specificity. But still, the study brings out the valuable knowledge and awareness in the fight against an increasing burden of end-stage renal disease (ESRD) for low-income countries. Another limitation was the inability of the researcher to track and interview none dialyzed ESRD patients who could have contributed new insights into resolving the problem. The researcher proposes comprehensive research which can utilize mixed-study design employing qualitative and quantitative approaches to be able to understand the depth and magnitude of the problem countrywide and elsewhere.

## Conclusion

The study clearly extrapolates that: non-medical facilitators are entirely confined to a small group of patients with health insurance and good family support. Patients with no health insurance and family support can receive chronic haemodialysis (CHD) services but not at the time of their need. Non-medical barriers which are beyond medical reasons impede most of the patients especially those residing outside Dar es Salaam and those who are financially poor from accessing chronic haemodialysis services (CHD). Ethical challenges identified are rapidly gaining momentum and seem to be far from being resolved. To avert challenging ethical issues and minimizing negative consequences of non-medical barriers and facilitators there is a need for a guideline highlighting the importance, position, and limitations of non-medical factors in the delivery of CHD services in dialysis units.

## Additional files

Additional file 1: Interview guides for both patients and healthcare providers in English and Swahili. (DOCX 24 kb)

## Abbreviations

CAPD: Continuous Ambulatory Peritoneal Dialysis
CHD: Chronic Haemodialysis
ESRD: End-stage Renal Disease
pmp: Per million population
