# Supplementary material for: Non-medical facilitators and barriers towards accessing haemodialysis services: an exploration of ethical challenges
Source: BMC Nephrol. 2018 Dec 3;19:342. doi: 10.1186/s12882-018-1140-x (PMC6276249; doi:10.1186/s12882-018-1140-x)
Supplement: Supplementary file 1 — Interview guides for both patients and healthcare providers in English and Swahili. (DOCX 24 kb) [file 12882_2018_1140_MOESM1_ESM.docx]

## INTERVIEW GUIDES FOR BOTH PATIENTS AND HEALTHCARE PROVIDERS

## Interview Guide for Health Care Providers [English Version]

**Introduction:** Thank you very much for agreeing to participate in this interview. The purpose of this study is to explore non-medical barriers and facilitators towards accessing chronic haemodialysis services at Muhimbili National Hospital dialysis unit and their ethical challenges. You have been selected among other service providers because you are knowledgeable about chronic haemodialysis services. During this interview there are no wrong answers, all answers are accepted so kindly feel free. I would like to remind you that this interview will take not more than 45minutes and it will be recorded to grab points that could be difficult to write down but also for easy analysis of the information later on.

**Qn1**. What non-medical factors do facilitate patients to access chronic haemodialysis services at this unit? How?

**Qn2**. What do you think are the non-medical barriers towards accessing chronic haemodialysis services for patients suffering from end-stage renal disease? Why?

**Qn3**. What ethical challenges do you as health care providers face in providing chronic haemodialysis services?

**Closure of the interview**: Is there anything else that you would like to share with me about what we have just discussed?

Thanks!

## Interview Guide for Patients [English Version]

**Introduction:** Thank you very much for agreeing to participate in this interview. The purpose of this study is to explore non-medical barriers and facilitators towards accessing chronic haemodialysis services at Muhimbili National Hospital dialysis unit and their ethical challenges. You have been selected among other patients because you are knowledgeable about chronic haemodialysis services. During this interview there are no wrong answers, all answers are accepted so kindly feel free. I would like to remind you that this interview will take not more than 45minutes and it will be recorded to grab points that could be difficult to write down but also for easy analysis of the information later on.

**Qn1**. What non-medical factors do facilitate access to chronic haemodialysis services at this unit? How?

**Qn2**. What do you think are the non-medical barriers towards accessing chronic haemodialysis services? Why?

**Qn3**. What ethical challenges do you face in accessing chronic haemodialysis services?

**Closure of the interview**: Is there anything else that you would like to share with me about what we have just discussed?

Thanks!

## Interview Guide for Health Care Providers [Swahili Version]

**Utangulizi:** Asante sana kwa kukubali kushiriki katika mahojiano haya. Utafiti huu una lengo la kuchunguza vikwazo visivyo vya kimatibabu na visaidizi vya kupata huduma za muda mrefu za usafishaji wa damu na changamoto zinazohusiana na maadili katika Hospitali ya Taifa ya Muhimbili. Umechaguliwa kati ya wahudumu wengine kwa sababu wewe ni mwenye ujuzi juu ya huduma ya usafishaji damu ya mda mrefu kwa wagonjwa wenye figo mfu. Katika mahojiano haya hakuna majibu yasiyo sahihi, majibu yote yanakubaliwa hivyo tafadhari jisikie huru. Napenda kukumbusha kwamba mahojiano haya hayata tumia zaidi ya dakika 45 pia yata nakiliwa na kinasa sauti ili kunyakua pointi ambazo inaweza kuwa vigumu kuandika lakini pia kwa ajili yakurahisisha uchambuzi wa mazungumzo haya apo badae.

**Swali la kwanza:** Unafikiri ni sababu zipi zisizo zakimatibabu uwezesha upatikanaji wa matibabu ya usafishaji damu kwa wagonjwa katika kitengo hiki? Kivipi?

**Swali la pili**: Je! Unafikiri ni vikwazo vipi visivyo vya kimatibabu upelekea au uzuia upatikanaji wa huduma ya usafishaji damu kwa mda mrefu kwa wagonjwa wa figo mfu? Kwa nini?

**Swali la tatu:** Je, ni changamoto gani za kimaadili ambazo ninyi kama watoa huduma za afya mnakabiliana nazo katika utoaji wa huduma za usafishaji damu wa mda mrefu?

**Kufungwa kwa mahojiano**: Je, kuna kitu kingine chochote ambacho ungependa kushiriki na mimi kuhusu mambo tuliyoyajadili?

Ahsante!

## Interview Guide for Patients [Swahili Version]

**Utangulizi:** Asante sana kwa kukubali kushiriki katika mahojiano haya. Utafiti huu una lengo la kuchunguza vikwazo visivyo vya kimatibabu na visaidizi vya kupata huduma za muda mrefu za usafishaji wa damu na changamoto zinazohusiana na maadili katika Hospitali ya Taifa ya Muhimbili. Umechaguliwa kati ya wagonjwa wengine kwa sababu wewe ni mwenye ujuzi juu ya huduma ya usafishaji damu ya mda mrefu kwa wagonjwa wenye figo mfu. Katika mahojiano haya hakuna majibu yasiyo sahihi, majibu yote yanakubaliwa hivyo tafadhari jisikie huru. Napenda kukumbusha kwamba mahojiano haya hayata tumia zaidi ya dakika 45 pia yata nakiliwa na kinasa sauti ili kunyakua pointi ambazo inaweza kuwa vigumu kuandika lakini pia kwa ajili yakurahisisha uchambuzi wa mazungumzo haya apo badae.

**Swali la kwanza:** Unafikiri ni sababu zipi zisizo zakimatibabu uwezesha upatikanaji wa matibabu ya usafishaji damu kwa wagonjwa katika kitengo hiki?

**Swali la pili**: Je! Unafikiri ni vikwazo vipi visivyo vya kimatibabu upelekea au uzuia upatikanaji wa huduma ya usafishaji damu kwa mda mrefu kwa wagonjwa wa figo mfu?

**Swali la tatu:** Je, ni changamoto gani za kimaadili ambazo unakabiliwa nazo katika kupata huduma za usafishaji damu wa mda mrefu?

**Kufungwa kwa mahojiano**: Je, kuna kitu kingine chochote ambacho ungependa kushiriki na mimi kuhusu mambo tuliyoyajadili?

Ahsante!
